# Supplementary material for: Reconciling Mining with the Conservation of Cave Biodiversity: A Quantitative Baseline to Help Establish Conservation Priorities
Source: PLoS One. 2016 Dec 20;11(12):e0168348. doi: 10.1371/journal.pone.0168348 (PMC5173368; doi:10.1371/journal.pone.0168348)
Supplement: S1 Dataset — (ZIP) [file pone.0168348.s002.zip › Taxa/Serra Norte/SN_2013/PES/Lista PESE-05.pdf]

CAVIDADE PESE-0005

Classe	Ordem	Fam/Outros	Gên/Outros	Espécie	S-U	U-S	Zon.
Arachnida	Acari	Macrochelidae		sp.2		X	P
Arachnida	Amblypygi	Phrynidae	*Heterophrynus*	*longicornis*		2	P
Arachnida	Araneae	Ochyroceratidae		jovem	X	X	P
Arachnida	Araneae	Oonopidae		jovem		X	E
Arachnida	Araneae	Pholcidae	*Mesabolivar*	*aurantiacus*	X	X	E/P
Arachnida	Araneae	Pisauridae		jovem	3	3	E/P
Arachnida	Araneae	Theridiosomatidae	*Plato*	sp.1	X	X	E/P
Arachnida	Opiliones	Escadabiidae		jovem	X		P
Arachnida	Opiliones	Gonyleptidae		jovem		X	P
Arachnida	Opiliones	Sclerosomatidae	*Prionostema*	sp.1	X	X	E
Arachnida	Pseudoscorpiones	Bochicidae		jovem	X		E
Entognatha	Collembola	Entomobryidae		sp.1	X		E
Entognatha	Collembola	Paronellidae		sp.1		X	E
Entognatha	Collembola	Paronellidae		sp.4	X		P
Entognatha	Collembola	Sminthuroidea		sp.1	X		E/P
Insecta	Blattodea			jovem	1		P
Insecta	Coleoptera	Carabidae		sp.22	X		E
Insecta	Coleoptera	Staphylinidae	Pselaphinae	sp.2	X	X	E/P
Insecta	Coleoptera	Staphylinidae	Pselaphinae	sp.42		X	P
Insecta	Coleoptera	Staphylinidae	Pselaphinae	sp.43		X	P
Insecta	Diptera	Ceratopogonidae		sp.	X		P
Insecta	Diptera	Drosophilidae	*Drosophila*	*eleonorae*		X	P
Insecta	Diptera	Ephydridae		sp.	X		P
Insecta	Diptera	Limoniidae		sp.	X		P
Insecta	Diptera	Psychodidae	*Pericoma*	sp.	X		P
Insecta	Diptera	Psychodidae	*Telmatoscopus*	sp.	X		P
Insecta	Diptera	Tipulidae	Tipulinae	sp.	X		E
Insecta	Heteroptera	Cydnidae	Cydninae	jovem		X	E
Insecta	Heteroptera	Veliidae	*Paravelia*	sp.1	X		E/P
Insecta	Hymenoptera	Formicidae	*Anochetus*	sp.1	X		E
Insecta	Hymenoptera	Formicidae	*Crematogaster*	sp.1	X		P
Insecta	Hymenoptera	Formicidae	*Nylanderia*	sp.3		X	E
Insecta	Hymenoptera	Formicidae	*Pheidole*	sp.2	X	X	E/P
Insecta	Hymenoptera	Formicidae	*Solenopsis*	sp.1	X		E
Insecta	Lepidoptera			jovem		X	P
Insecta	Orthoptera	Phalangopsidae	*Phalangopsis*	sp.	35	64	E/P
Insecta	Psocoptera	Psocomorpha		jovem		X	E
Malacostraca	Decapoda	Trichodactylidae		sp.	1		E
Malacostraca	Isopoda	Scleropactidae		sp.1	X		P
Amphibia	Anura	Strabomantidae	*Pristimantis*	cf. *fenestratus*	1		P
Reptilia	Squamata	Gymnophthalmidae	aff. *Neusticurus*	sp.		2	P
